# Real-Time fMRI in Neuroscience Research and Its Use in Studying the Aging Brain

**DOI:** 10.3389/fnagi.2016.00239

**Published:** 2016-10-18

**Authors:** Mohit Rana, Andrew Q. Varan, Anis Davoudi, Ronald A. Cohen, Ranganatha Sitaram, Natalie C. Ebner

**Affiliations:** ^1^Department of Psychiatry and Division of Neuroscience, School of Medicine, Pontificia Universidad Católica de ChileSantiago, Chile; ^2^Laboratory for Brain-Machine Interfaces and Neuromodulation, Pontificia Universidad Católica de ChileSantiago, Chile; ^3^Department of Psychology, University of FloridaGainesville, FL, USA; ^4^J. Crayton Pruitt Family Department of Biomedical Engineering, University of FloridaGainesville, FL, USA; ^5^Center for Cognitive Aging and Memory, Institute on Aging, University of FloridaGainesville, FL, USA; ^6^Department of Aging and Geriatric Research, College of Medicine, University of FloridaGainesville, FL, USA; ^7^Institute for Biological and Medical Engineering, Schools of Engineering, Biology and Medicine, Pontificia Universidad Católica de ChileSantiago, Chile

**Keywords:** real-time functional magnetic resonance imaging, aging, cognition, neurofeedback, emotion

## Abstract

Cognitive decline is a major concern in the aging population. It is normative to experience some deterioration in cognitive abilities with advanced age such as related to memory performance, attention distraction to interference, task switching, and processing speed. However, intact cognitive functioning in old age is important for leading an independent day-to-day life. Thus, studying ways to counteract or delay the onset of cognitive decline in aging is crucial. The literature offers various explanations for the decline in cognitive performance in aging; among those are age-related gray and white matter atrophy, synaptic degeneration, blood flow reduction, neurochemical alterations, and change in connectivity patterns with advanced age. An emerging literature on neurofeedback and Brain Computer Interface (BCI) reports exciting results supporting the benefits of volitional modulation of brain activity on cognition and behavior. Neurofeedback studies based on real-time functional magnetic resonance imaging (rtfMRI) have shown behavioral changes in schizophrenia and behavioral benefits in nicotine addiction. This article integrates research on cognitive and brain aging with evidence of brain and behavioral modification due to rtfMRI neurofeedback. We offer a state-of-the-art description of the rtfMRI technique with an eye towards its application in aging. We present preliminary results of a feasibility study exploring the possibility of using rtfMRI to train older adults to volitionally control brain activity. Based on these first findings, we discuss possible implementations of rtfMRI neurofeedback as a novel technique to study and alleviate cognitive decline in healthy and pathological aging.

## Introduction

### Age-Related Cognitive Decline and Underlying Brain Mechanisms

Given current demographic developments with adults over the age of 65 years representing the fastest growing segment of the population in the USA and other industrialized nations (Census, 2012), cognitive decline in aging is of increasing societal and economic relevance, in addition to its relevance to individual lives (Williams and Kemper, 2010). It is usual, with interindividual variation (Ram et al., 2011), to experience some deterioration in cognitive abilities with advanced age. These age-related cognitive deficits are typically characterized by slow processing speed (Eckert et al., 2010), increased difficulty in encoding and retrieving memories (Wilckens et al., 2012), increased forgetfulness (Gazzaley et al., 2005), reduced ability to selectively attend to or ignore irrelevant information (Prakash et al., 2009), increased distraction to interference (Wais et al., 2012), and reduced task switching abilities (Buchler et al., 2008). This change in cognitive functioning constraints older adults’ independence and quality of life (Logsdon et al., 2002). Thus, studying ways to counteract or delay the onset of cognitive decrement in aging is crucial.

Recent research initiatives address cognitive decline and brain aging, such as the “Healthy Brain Initiation” by the American Association of Retired Persons^1^ and the Alzheimer’s Association^2^ in the US, and the “Healthy Brain” initiative by the Brain Foundation^3^ in Australia. These initiatives have targeted the creation of standardized assessment tools and the implementation of lifestyle directives (e.g., related to nutrition and physical activity) to allow for direct comparison across research studies and to inform interventional strategies towards maintenance and promotion of cognitive functioning in older adults or delay of cognitive decline until later in life. There is also a growing market for computer-based trainings, memory tapes, and computer games offered to the lay public. These products claim enhancement of cognitive performance through training (Casel, 2002). However, most of these current approaches target training of behavioral aspects of cognitive aging without consideration of brain processes.

With the recent advancement in neuroimaging technology, and especially developments in functional magnetic resonance imaging (fMRI), understanding of functional brain changes that underlie age-related cognitive decline has tremendously increased (Li et al., 2015). For instance, aging has been shown to be associated with greater involvement of frontal and parietal regions and reduced activation of occipital regions during attention (Cabeza et al., 2004), visual perception (Davis et al., 2008), working memory (Park et al., 2003), language (Grossman et al., 2002), and emotion processing (Williams et al., 2006). These findings have been discussed in the context of proposed models of brain aging, such as the hemispheric asymmetry reduction in older adults (HAROLD; Cabeza, 2002), the posterior–anterior shift in aging (PASA; Davis et al., 2008), the compensation-related utilization of neural circuit hypothesis (CRUNCH; Reuter-Lorenz and Cappell, 2008), and the scaffolding theory of aging and cognition (STAC; Goh and Park, 2009). According to the HAROLD model, older compared to younger adults show greater bilateral brain activity in prefrontal cortex (PFC) for specific cognitive tasks. Similarly, the CRUNCH model proposes that older adults have lower neural efficiency than younger adults. That is, older compared to younger adults recruit more brain regions (e.g., frontal or bilateral brain regions) for cognitive operations. The PASA model of aging states that older adults during task engagement show increased activations of PFC coupled with decreased activation of the occipital cortex leading to a shift in brain activity pattern from the posterior part of the brain to the anterior part. Similarly, STAC suggests that an increasing use of frontal brain regions with age during cognitive processing is an indication of an adaptive brain. The theory proposes that, to counter the deterioration of neural structures and functions with age, the brain develops compensatory neural circuits to achieve a particular cognitive goal.

An independent emerging literature has generated exciting results that support the benefits of volitional modulation of activity in specific brain regions and networks on cognition and behavior. In particular, a number of studies have shown that individuals can learn to voluntarily control different components of the electroencephalographic (EEG) spectrum, resulting in specific behavioral change (Kotchoubey et al., 2001; Kubler et al., 2001; Fuchs et al., 2003; Murase et al., 2004; Birbaumer, 2006; Strehl et al., 2006). EEG based neurofeedback has advantages of high temporal resolution, affordability and portability, but it has disadvantages related to its low spatial resolution, and its inability to access deeper brain regions. It also suffers from computational complexity of the inverse problem in determining the source of activations from the surface EEG signals. However, recent developments in real-time fMRI (rtfMRI; Weiskopf, 2012; Sulzer et al., 2013; Stoeckel et al., 2014) have overcome some of the limitations of the EEG based technique due to fMRI’s high spatial resolution and its capacity for whole brain coverage.

In this article, we offer a state-of-the-art description of the neurofeedback technique with a particular focus on rtfMRI in its application to cognitive aging. To the best of our knowledge, no research to date has used rtfMRI in the context of studying and counteracting cognitive decline in older adults. We start by outlining current empirical evidence on cognitive and behavioral benefits of rtfMRI in young healthy adults as well as patients. We then discuss the application of this novel technique in the context of aging research, supported by preliminary data from our group. The article concludes with a discussion of future research directions using rtfMRI and related neurofeedback training techniques, such as EEG and functional near-infrared spectroscopy (fNIRS), towards preservation of cognitive function and delay of cognitive decline in aging.

### Volitional Modulation of Brain Activity via rtfMRI

Neurofeedback is a procedure by which humans or animals can learn to modulate neural activity in one or more brain region(s) (Birbaumer et al., 2013). The mechanism underlying neurofeedback learning is still not completely understood, but several different mechanisms, including, operant conditioning and skill learning, have been proposed (Sitaram et al., in press). For example, via rtfMRI neurofeedback training, volitional increase or decrease of Blood Oxygenation Level Dependent (BOLD) response in a circumscribed brain area or network of regions can be attained by the subjects.

### Overview of the rtfMRI System

**Figure 1:**
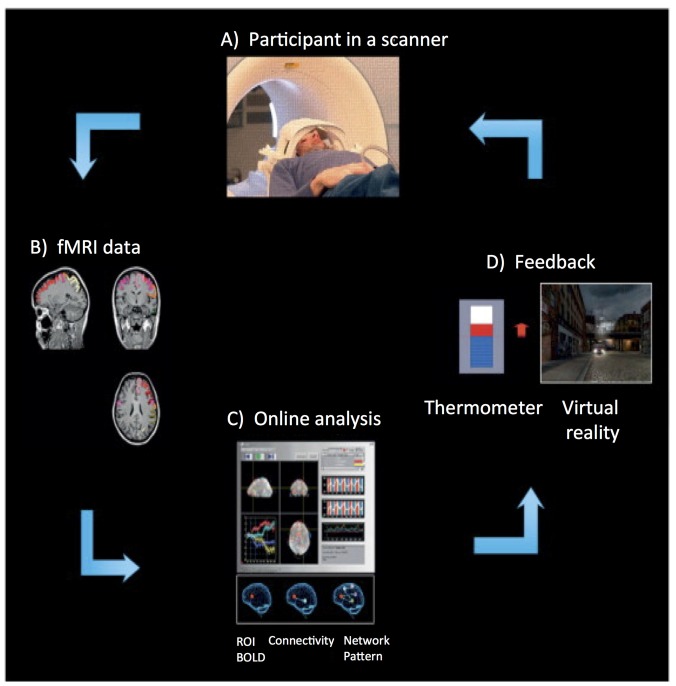
**Overview of an real-time functional magnetic resonance imaging (rtfMRI)-based neurofeedback system that comprises the following subsystems. (A)** Participant in the MRI scanner. **(B)** Signal acquisition (fMRI data) using an echo planar imaging (EPI) pulse sequence. **(C)** Online analysis and computation of the neurofeedback based on the Blood Oxygenation Level Dependent (BOLD) response. **(D)** Visual feedback via the scanner projection system. This figure is adapted from Birbaumer et al. (2013). The rtfMRI system presented in this figure was developed at the Institute of Medical Psychology and Behavioral Neurobiology, University of Tübingen, Germany.

More than a decade ago, the first fMRI based Brain Computer Interface (BCI) approach was implemented (Posse et al., 2003). Figure 1 depicts the rtfMRI neurofeedback system. It is a closed-loop system that uses the BOLD signal from one circumscribed brain region or a network of brain regions, in real-time, to calculate and present feedback (e.g., visual, auditory, or tactile) to participants (e.g., Caria et al., 2007; Sitaram et al., 2007; Rota et al., 2009; Ruiz et al., 2013a). The rtfMRI system comprises of the following subsystems (see Figure 1): (A) participant, (B) signal acquisition, (C) online signal analysis, and (D) feedback.

An echo planar imaging (EPI) sequence (Bandettini et al., 1992) is used to acquire functional images of the brain (see Figure 1B). Online computation procedures with the data in the k-space such as distortion correction, averaging of the signal, and image reconstruction are performed on the scanner’s image reconstruction computer. Once the image is reconstructed and pre-processed, it is exported to the signal analysis subsystem (see Figure 1C). The signal analysis subsystem is implemented using the Turbo Brain Voyager (TBV) software (Brain Innovations, Maastricht, Netherlands). TBV retrieves the reconstructed image and performs data processing that includes 3D motion correction and real-time statistical analysis using the general linear model. TBV allows the user to draw regions of interest (ROIs) on the functional images. The BOLD values pertaining to these ROIs are exported to a Matlab script (Mathworks, Natwick, MA, USA) that calculates the feedback, which is then presented to the participant inside the scanner (see Figure 1D).

Diverse modalities of feedback can be employed, including verbal, auditory, tactile, monetary, or a combination of these, but visual feedback has been predominantly used in research. Visual feedback of the brain activity can be provided to the participant in the form of a graphically animated thermometer with bars of the thermometer changing in proportion to the percent BOLD changes in the ROIs. The majority of rtfMRI studies reported in the literature applied continuous feedback (i.e., feedback provided to a participant within one repetition time, TR, of the EPI sequence). For example, Caria et al. (2007) showed that participants were able to self-regulate anterior insula when trained with continuous feedback with a delay of 1 TR (i.e., 1.5 s), while participants who received sham feedback did not learn to self-regulate. This finding demonstrated the importance of contingent feedback to learn to self-regulate neural activity. However, intermittent feedback (i.e., feedback provided to a participant after a number of TRs of the EPI sequence) has also been used successfully (Yoo and Jolesz, 2002; Johnson et al., 2012).

### Typical Design of rtfMRI Studies

A typical rtfMRI study consists of a number of neurofeedback training sessions, in which a participant learns to regulate (increase or decrease) the BOLD signal in a particular ROI. Typically, a neurofeedback training run consists of two types of conditions, namely baseline and regulation, although there is no general rule in this regard. In the majority of current studies, participants were instructed to remain in a resting state during the baseline blocks and to find a cognitive strategy that helps them to achieve self-regulation in the regulation blocks. However, there are also studies where participants were not given any instructions to use a cognitive strategy, but were trained with just real-time feedback or reward (Shibata et al., 2011; Sepulveda et al., 2016).

### Types of rtfMRI Neurofeedback Approaches

The current literature generally differentiates between three types of rtfMRI neurofeedback approaches, namely, single-ROI based neurofeedback (Caria et al., 2007), functional connectivity based neurofeedback (Liew et al., 2015) and network pattern based neurofeedback (Shibata et al., 2011). Development of these approaches occurred independent of each other and temporally overlapped. The suitable rtfMRI neurofeedback approach for a specific study is selected based on the study’s hypothesis.

#### Single-ROI Based Neurofeedback

Single-ROI based neurofeedback approach is a more conservative rtfMRI approach than the other two approaches. In this approach, individuals learn to volitionally regulate the BOLD signal from one circumscribed brain area. Feedback is calculated as a linear combination of the signal amplitude in the target ROI (e.g., motor cortex for a motor task) and a task-unrelated reference ROI (e.g., auditory cortex for a motor task). The reference area is used to subtract the global (whole-brain) increase in the BOLD signal due to general arousal, task-unrelated factors, or BOLD fluctuations caused by head motion. An example equation (Equation 1) for calculating feedback using this approach is as follows:

(1) $$\begin{array}{l} \text{Feedback}\;=\;(\text{ROI}1_{\text{Regulation}}-\text{ROI}1_{\text{Baseline}})\\ \;-(\text{ROI}2_{\text{Regulation}}-\text{ROI}2_{\text{Baseline}}) \end{array}$$

where ROI1 is the target brain area and ROI2 is the reference brain area.

#### Functional Connectivity Based Neurofeedback

In general, a brain function can hardly be conceived to involve only one single brain region (Sporns et al., 2005). Rather, the brain is considered to work by coordinating activity across distributed brain regions to execute a task. Functional connectivity is defined as the statistical dependency between two or more remote neurophysiological events (Friston, 2011). It represents the connectivity between two or more brain regions that share functional properties. There are two methods of computing functional connectivity. The first method involves estimation of statistical correlation between the BOLD time-series of two ROIs using the Pearson, sample, or population correlation coefficient. Functional connectivity neurofeedback using this first method can be calculated either by including the correlation measure in the standard ROI feedback equation (e.g., Equation 2; Ruiz et al., 2012), or by subtracting correlations of the two ROIs in the baseline condition from those in the regulation condition (e.g., Equation 3; Liew et al., 2015).

(2) $$\begin{array}{l} \text{Feedback}\;=\;(\text{TOT}\_{\text{BOLD}}_{\text{Regulation}}-{\text{TOT}}_{\text{BOLD}}{}_{\text{Baseline}})\\ \;\times (1+\text{EC}) \end{array}$$

(3) $$\text{Feedback}\;=\;{\text{EC}}_{\text{Regulation}}-{\text{EC}}_{\text{Baseline}}$$

where TOT_BOLD is the total BOLD signal in the two ROIs (i.e., TOT_BOLD = (BOLD in ROI1 + BOLD in ROI2) and EC is the Pearson’s linear correlation coefficient derived from the BOLD time-series of these two ROIs.

A sliding window of eight data points, i.e., the current time point and seven data points before the current data point from each ROI, is used to compute the correlation coefficient. The limitation of this method is that it does not provide any information about the causality or the direction of information flow, between the two ROIs. This limitation is addressed by the second method of computing functional connectivity, in that it estimates effective connectivity using Granger causality (GC; Granger, 1969) or dynamic causal modeling (DCM; Friston et al., 2003) There is evidence that use of DCM allows training of effective connectivity between two ROIs to influence the directionality of functional interactions (Koush et al., 2013).

#### Network Pattern Based Neurofeedback

Compared to the single-ROI based or the functional connectivity based approaches, the pattern classification approach provides greater sensitivity for detection and modulation of an entire brain network involved in a specific function (Haynes and Rees, 2006; Lewis-Peacock and Norman, 2014; Haynes, 2015). In this approach, spatial and temporal patterns of activity of multiple brain regions involved in a function are computed in real-time and presented as feedback to the participant. There are several different pattern classification techniques that have been applied to fMRI data, including Linear Discriminant Analysis (LDA; LaConte et al., 2003), Naïve Bayes (Pereira et al., 2009), Support Vector Machine (SVM; LaConte et al., 2005), Neural Networks (Hanson et al., 2004), Canonical Variates Analysis (Mourão-Miranda et al., 2006), and Fisher Linear Discriminant (Shaw et al., 2003). SVM is one of the methodologies widely used to predict the brain state based on the BOLD signal. There is evidence that SVM provides higher classification accuracy than the other methods of pattern classification (LaConte et al., 2003, 2005; Shaw et al., 2003; Strother et al., 2004; Martínez-Ramón et al., 2006). SVM is less sensitive to preprocessing steps when compared with LDA (i.e., high classification accuracy), which is useful in real-time applications (LaConte et al., 2005). SVM is a binary classification algorithm that estimates a hyper-plane in a multi-dimensional space to discriminate between two tasks (Schölkopf and Smola, 2002). BOLD signals from two different conditions (e.g., left and right hand movement imagery) are fed into the SVM algorithm and a classification model is generated based on this information. Based on this generated classification model, SVM predicts the possible condition that a particular preprocessed BOLD signal belongs to.

There are currently two approaches of performing the online pattern classification: subject-dependent classification and subject-independent classification (Rana et al., 2013). The majority of current studies used the subject-dependent classification approach, in which classification models are tailored to a specific participant’s brain signals. Some studies have shown impressive results using this approach in healthy adults (Shibata et al., 2011; Sitaram et al., 2011; deBettencourt et al., 2015). However, this technique only led to very limited advancement in the field of neurorehabilitation. The main reason for this limitation is that classification models are not sufficiently generic to be used across participants due to interindividual variations in structural and functional brain characteristics, which may even be exaggerated in clinical population and may become more pronounced in aging (Meunier et al., 2014). This limitation of the subject-specific classification constitutes a hindrance for its application in patients and older adults. In contrast, the subject-independent classifier approach can be applied to healthy as well as patient populations without the need to collect subject-specific data to generate the classifier model. In addition, it can be adapted to the idiosyncrasies of individual brain size, shape, and activation patterns. Thus, the subject-independent approach has the potential to facilitate training of patients to correct and tune their abnormal brain activity towards normalcy and appears to have promise for applications in aging.

### Evidence for Cognitive and Behavior Modification Using rtfMRI Neurofeedback

The rtfMRI neurofeedback approach represents a new tool for studying the relation between brain activity, cognition, and behavior. Importantly, unlike in conventional neuroimaging approaches where cognition and behavior are the independent variables and brain activity is the dependent variable, in rtfMRI, brain activity constitutes the independent variable while cognition and behavior serve as the dependent variables. Neurofeedback using rtfMRI has been used to train individuals in volitional regulation of BOLD signals in different brain regions or in connectivity among multiple regions, to determine cognitive and behavioral effects of learned self-regulation. In this endeavor, neurofeedback effects have been documented in pain modulation, in reaction times, and linguistic and emotional processing in young healthy and/or patient populations (deCharms et al., 2005; Rota et al., 2009; Ruiz et al., 2013a; Scharnowski et al., 2015).

In the early days of rtfMRI, studies were focused on evaluating effects due to self-regulation in a single, circumscribed brain area (i.e., single-ROI neurofeedback approach). This nascent field originally applied, and attempted to optimize, this more parsimonious methodology. A number of studies were conducted using volitional brain regulation of single areas such as the insula, amygdala, visual cortex, anterior cingulate, or motor cortex to understand their impact on cognition, perception, and emotion (e.g., Caria et al., 2010; Shibata et al., 2011; Paret et al., 2014; Gröne et al., 2015). For example, Posse et al. (2003) conducted the first rtfMRI study in emotion-related brain areas. Participants were trained to modulate amygdala activity using a self-inducing mood paradigm that included sad and neutral emotional states. All participants in this study were able to successfully achieve sad mood. Further, mood self-ratings were positively associated with BOLD response in amygdala. However, in this study, the self-induction task was performed in the presence of emotional faces during the entire experiment and the study lacked a control group (CG). Thus, it was not clear whether the observed correlation was due to the amygdala self-regulation or due to the presentation of emotional faces, or a combination of both. Caria et al. (2010) observed significant modification in valence ratings related to aversive picture stimuli associated with up-regulation of anterior insula in individuals trained with contingent feedback, while the effect was not observed in individuals trained with sham feedback. This study provided further support for cognitive and behavioral modification induced by learned self-regulation and the importance of contingent feedback in neurofeedback training.

Some rtfMRI studies examined cognitive and behavioral effects using functional connectivity based neurofeedback. This was based on the rationale that a brain function works via coordination of distributed brain regions to execute a task. This development was also informed by emerging evidence that abnormal connectivity of brain areas was associated with abnormal brain functioning in neuropsychiatric disorders such as schizophrenia (Friston and Frith, 1995; Honey et al., 2005), autism (Just et al., 2007), Alzheimer’s Disease (AD; Wang et al., 2007; Zhang et al., 2010), and Attention Deficit Hyperactivity Disorder (ADHD; Konrad and Eickhoff, 2010). Use of the connectivity approach was also spurred by evidence that learned volitional control of a single brain area in healthy adults lead to changes in functional connectivity across brain regions (Hamilton et al., 2011; Lee et al., 2011; Zotev et al., 2011; Ruiz et al., 2013a,b). For example, a study in schizophrenia patients found that learned self-regulation of a single brain ROI modulated brain connectivity in an entire network (Ruiz et al., 2013b), supporting the use of rtfMRI as a tool to enhance brain connectivity. However, enhancement of functional connectivity between various brain areas in this early study was observed as a by-product of single-ROI based neurofeedback training but was not the result of direct training of brain connectivity.

Thus, following up on these initial findings, studies using rtfMRI based connectivity neurofeedback demonstrated that enhancement of functional connectivity between two brain areas was possible and resulted in cognitive and behavioral modifications. Kim et al. (2015), for example, showed improved efficacy in reducing cigarette smoking via learned self-regulation of connectivity between four brain regions related to craving (i.e., anterior cingulate cortex, medial PFC, posterior cingulate cortex, and precuneus). Similarly, a study from our lab found evidence for direct enhancement of brain functional connectivity (Ruiz et al., 2012). In particular, healthy adults were trained to increase functional connectivity between inferior frontal gyrus (Broca’s area) and superior temporal gyrus (Wernicke’s area), which resulted in an enhanced priming effect in a semantic priming task (Sass et al., 2009).

In parallel to the other two neurofeedback approaches, the field explored the use of rtfMRI in the context of pattern classification based neurofeedback. The first study used SVM algorithm for binary classification in the context of real-time feedback on motor and cognitive states (LaConte et al., 2007). Sitaram et al. (2011) implemented a mapping technique for pattern classification of multiple emotional brain states in real-time. Shibata et al. (2011) demonstrated perceptual learning by inducing spatial patterns of activity in the primary visual cortex. In this study, pattern feedback was used to train participants to self-induce brain activity pertaining to one of three Gabor patch gratings (differing by 60° from one another), without participants’ awareness of the target grating. Behavioral data collected after the neurofeedback training showed improved sensitivity to the target grating as compared to the other two gratings. This finding suggested that induction of activity patterns in the primary visual areas was sufficient for perceptual learning. A study by deBettencourt et al. (2015) further showed improved sustained attention and reduced frequency of lapses in attention using closed-loop neurofeedback. Neurofeedback was provided to participants based on their level of attention to pictures of faces and scenes in a Go-NoGo task. Task difficulty was anti-correlated with the level of attention detected by the pattern classification algorithm. Pattern classification algorithm captured a widely distributed network of brain activity associated with top-down attentional control. The same pattern of activity was enhanced by neurofeedback training, which improved participants’ attentional vigilance.

The above brief review of the current literature on rtfMRI demonstrates the potential that this technique has to study cognitive and behavioral modulation via brain activation. That is, learned self-regulation of a brain region, functional connectivity of two brain areas, or a network of brain areas can serve as independent variables in determining the effect of volitional control on cognition and behavior. Our summary also highlights the promise this novel approach offers for brain-behavior interventions. Importantly, to date, this exciting new tool has not yet been applied to aging research. In the remaining sections of this article we show that the use of rtfMRI in older adults is feasible and we propose that it constitutes a powerful technique to study cognitive function and the aging brain.

### Feasibility of Applying rtfMRI Neurofeedback in Aging Research

We conducted a pilot study to determine the feasibility of using rtfMRI neurofeedback in research with older adults. In particular, we examined a neurofeedback training scheme in the context of an emotion perception paradigm in a sample of eight adults aged 61 years and older. Our study was based on evidence that aging is associated with emotional changes (Ebner et al., 2006; Blanchard-Fields, 2007; Scheibe and Carstensen, 2010; Ebner and Fischer, 2014). For example, apathy increases with age and is associated with cognitive decline (Brodaty et al., 2010) and constitutes one of the central causes of suffering of close relatives (Benoit et al., 2008) leading to poor quality of life (Yeager and Hyer, 2008). Also, aging is accompanied by less effective use of some emotion-regulatory strategies (Winecoff et al., 2011; Opitz et al., 2012), and increased difficulty in the perception of emotions in others (Ruffman et al., 2008; Ebner et al., 2010). This age-related decline has the potential to negatively impact emotional well-being and quality of social relationships (Ruffman et al., 2012), putting older adults at increased risk for social isolation and reduced health (Cornwell and Waite, 2009; Norman et al., 2011). Research suggests that alterations in brain function associated with affective processing contribute to these emotional changes in aging (Williams et al., 2006; Samanez-Larkin and Carstensen, 2011; Winecoff et al., 2011; Ebner et al., 2012).

Our study particularly focused on volitional control of the anterior insula, a region in the limbic system that is crucially involved in affective processing such as evaluation and arousal (Berntson et al., 2011). Anterior insula function appears to be impacted by aging. In particular, there is evidence of dampened anterior insula activity in older adults, with effects on affective processing (Castle et al., 2012). Previous rtfMRI studies have shown that it is possible to train young adults (Caria et al., 2007, 2010) and schizophrenia patients (Ruiz et al., 2013a) to self-regulate anterior insula with contingent feedback, modulating affective processing.

Based on this evidence, we aimed to examine whether older adults could learn to self-regulate anterior insula with contingent rtfMRI neurofeedback and whether learned self-regulation could lead to behavioral modification in this age group. We used a single-ROI rtfMRI approach to train older adults to either up-regulate anterior insula (experimental group; EG) or primary auditory cortex (CG), a brain region not specifically associated with affective processing (Pavuluri et al., 2007; Tracy and Robins, 2008). Eight older adults (mean age: 66 ± 5.18 years; five women) participated in the study. Five participants were randomly assigned to the EG and three to the CG. The experiment consisted of six rtfMRI sessions conducted over a period of a couple of weeks. In the first and the last rtfMRI session, participants engaged in a facial emotion recognition task (for similar paradigms, see Ebner and Johnson, 2009; Ebner et al., 2012). Each participant undertook 18–20 neurofeedback training runs distributed among four rtfMRI based neurofeedback training sessions.

**Figure 2:**
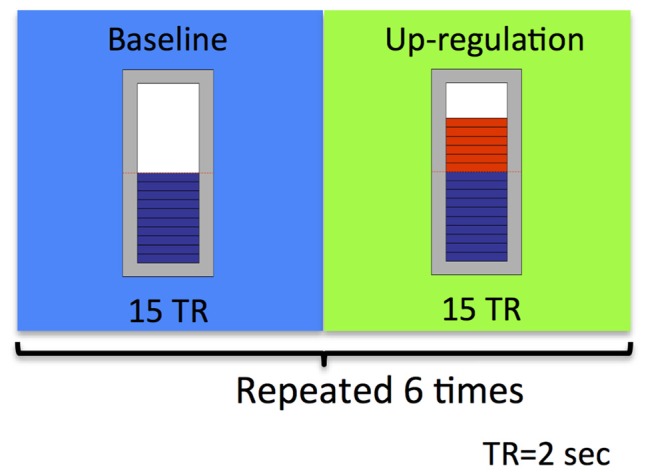
**Schematic illustration of the experimental neurofeedback paradigm used in our feasibility study.** An rtfMRI-based neurofeedback training run consisted of alternating baseline and up-regulation blocks, repeated six times. Each block was 30 s long.

The study took place at the Advanced Magnetic Resonance Imaging and Spectroscopy (AMRIS) facility of the McKnight Brain Institute (MBI), where brain imaging was conducted on a 3.0 Tesla, 32-channel Philips whole-body human MR scanner. As depicted in Figure 2, an rtfMRI based neurofeedback training run consisted of alternating baseline and up-regulation blocks, each lasting 30 s. There were six up-regulation and six baseline blocks in total per run. Participants were suggested to use imagery to recall emotionally relevant experiences. Neurofeedback was provided to them visually in the form of a thermometer. During up-regulation blocks, the graphical thermometer was presented over a green background and the bars in the thermometer were changed based on the up-regulation of BOLD signal that the participant achieved relative to the BOLD signal in the preceding baseline block. Increase in the number of bars of the thermometer represented more successful up-regulation performance by the participant. During the baseline block, the feedback bar remained stationary over a blue background.

**Figure 3:**
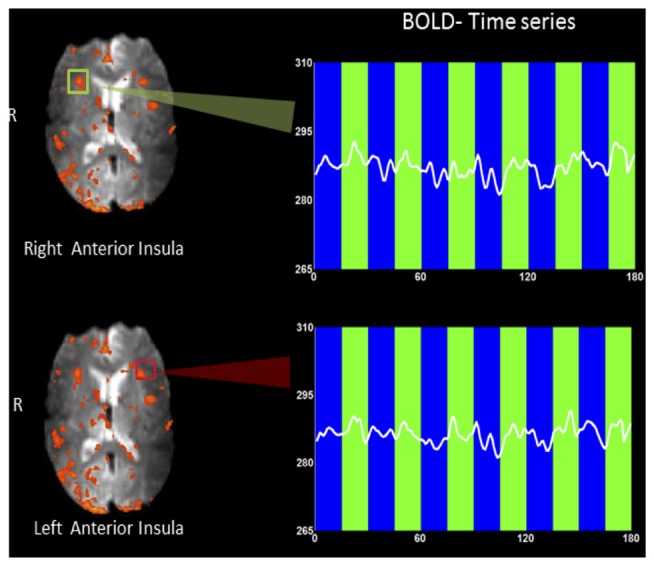
**An example rtfMRI run showing the up-regulation of BOLD response in the predetermined regions of interest (ROIs) of right (green rectangle) and left (red rectangle) anterior insula, overlaid on a mean EPI brain image.** Mean BOLD signals were extracted from each of the two ROIs and are presented overlaid on baseline (blue bars) and up-regulation (green bars) blocks. A moderate increase in BOLD signal in both left and right insula (higher amplitude) was observed during regulation blocks as compared to baseline blocks.

Figure 3 shows an example rtfMRI run in a single subject showing the up-regulation of BOLD response in the right and left anterior insula, overlaid on an average EPI brain image. A moderate increase in BOLD signals in both left and right insula was observed during regulation blocks as compared to baseline blocks. ROI analysis (Poldrack, 2007) was conducted using BOLD values extracted from two rectangles, each of size 5 × 5 voxels (~15 mm^2^ × 15 mm^2^).

**Figure 4:**
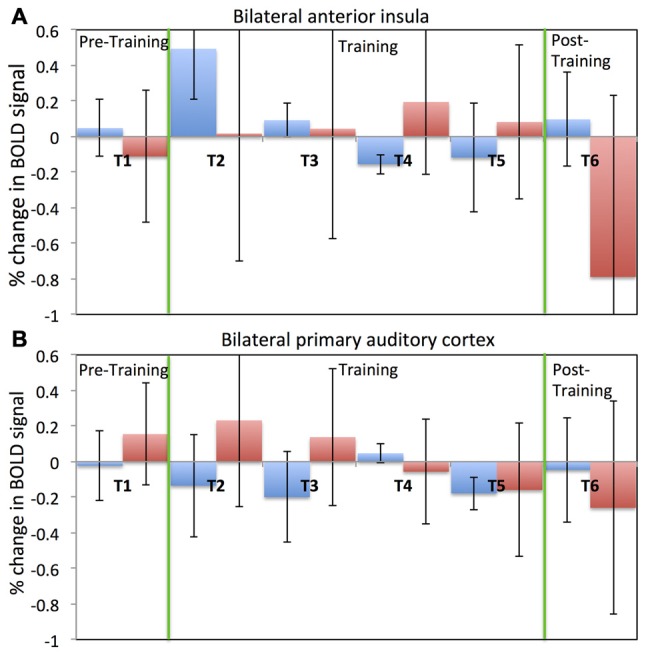
**Percentage change in BOLD signal for each run across all six fMRI sessions.** Average percentage change in bilateral anterior insula cortex **(A)** for both the experimental (blue) and the control (red) groups. Average percentage change in bilateral primary auditory cortex **(B)** for both the experimental (blue) and the control (red) groups. Percentage change in the BOLD signal is calculated by comparing change in the BOLD signal during the regulation block to the previous baseline block. A positive value of the percentage change in BOLD signal indicates up-regulation of BOLD signal.

Figure 4 depicts results regarding older adults’ ability to self-regulate a circumscribed brain area with contingent neurofeedback training. Participants in both the EG (see Figure 4A) and the CG (see Figure 4B) were able to up-regulate activity in anterior insula and primary auditory cortex, respectively, in the initial neurofeedback training sessions as reflected in positive values of the percentage change in the BOLD signal. Participants in the EG were able to achieve up-regulation of BOLD signal in the anterior insula for the first two training session (T2 and T3). However, their performance diminished in the fourth and fifth training session (T4 and T5). In the last session (T6), participants in the EG were able to up-regulate in six out of eight rtfMRI runs. A somewhat similar pattern of findings was observed in the CG (see Figure 4B): CG participants were able to up-regulate BOLD signal in the primary auditory cortex in the first two training sessions (T2 and T3). However, in later sessions, control participants were not able to maintain their performance.

**Figure 5:**
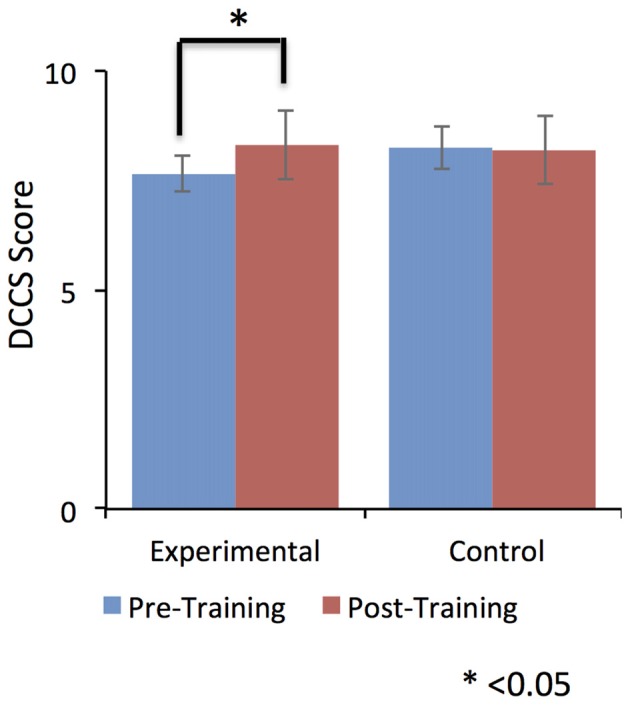
**Dimensional change card sort (DCCS) scores were significantly increased from pre-training to post-training in the experimental group (EG).** No effect was observed for the control group (CG). This result suggests a positive effect of self-regulation of bilateral anterior insula on older adult’s cognitive flexibility. Error bars represent the standard deviation in DCCS scores.

Despite this dip in performance in the last two training sessions, participant in the EG (but not participants in the CG) performed relatively better in the post-training session compared to the pre-training session. Further, this self-regulation training lead to behavioral modification. In particular, there was an effect on participants’ cognitive flexibility in the EG, but not in the CG. Cognitive flexibility was measured with the dimensional change card sort test (DCCS; Zelazo, 2006) from the cognitive test battery in the NIH toolbox (Heaton et al., 2014). As shown in Figure 5, the EG but not the CG showed a significant increase (*T* = −3.9, one tailed; *p* = 0.008) in cognitive flexibility post-training (8.31 ± 0.8) compared to pre-training (7.6 ± 0.63). This behavioral modification was specific to self-regulation training of the anterior insula, as it was not observed in the auditory cortex CG.

**Figure 6:**
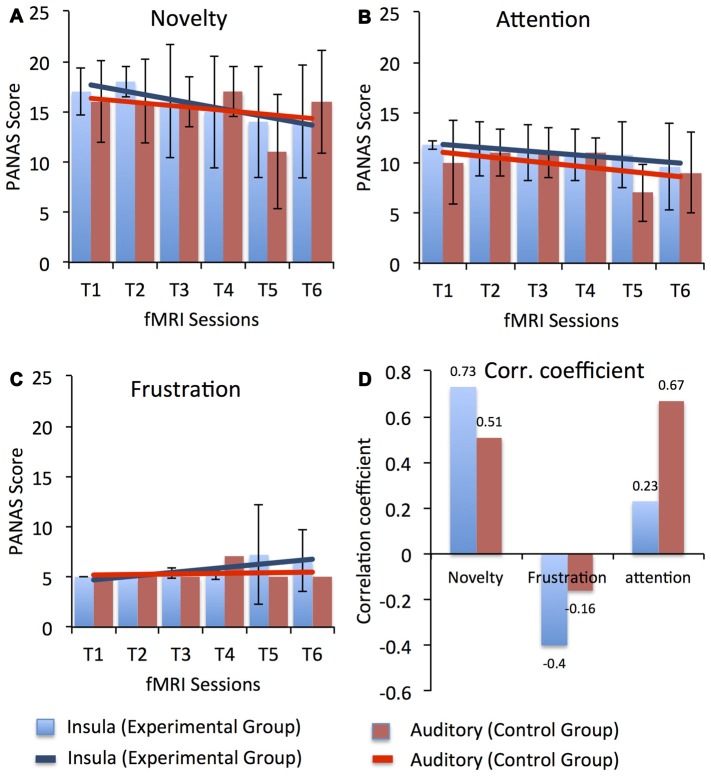
**Average Positive Affect and Negative Affect Scale (PANAS) score for three different aspect of participants’ current mood with possible relevance to the learning process, namely, novelty/motivation (comprised the adjectives interested, excited, inspired, enthusiastic, determined), attention (comprised the adjectives alert, attentive, active) and frustration (comprised the adjectives distressed, downhearted, upset, frustrated, irritable) across all fMRI sessions for both the experimental (blue) and the control (red) groups.** Scores for novelty, attention and frustration were calculated by averaging across the rating of respective adjectives. Error bars represent the standard deviation in the scores for novelty, attention and frustration across the group. Results from a linear regression analysis are represented by the solid blue and red lines for the experimental and the CGs, respectively. A gradual linear downward trend was observed in the ratings of novelty **(A)** and attention **(B)** across the sessions in both groups. However, a minute increase was observed in the ratings of frustration **(C)** in the later sessions for participants in both groups. High positive correlation coefficients **(D)** between up-regulation performance (i.e., % change in BOLD signal in bilateral anterior insula for the EG and in bilateral primary auditory cortex for the CG) and the PANAS scores were observed for novelty and attention, indicating that reduced motivation and attention levels might have led to reduced self-regulation performance. In contrast, a negative correlation was observed between PANAS scores for frustration and up-regulation performance in both groups, indicating that frustration did not affect participants’ self-regulation performance.

Possible factors that could have led to the dip in self-regulation performance observed in our study participants during the course of the training may have been related to the dual-task conflict inherent in the approach or increased fatigue as the study progressed. During neurofeedback training, to achieve self-regulation, participants have to apply a cognitive strategy to get positive feedback and simultaneously they have to evaluate the feedback presented to them. Therefore, participants have to switch between two tasks, which is cognitively demanding, particularly for older adults. Factors related to motivation and attention could also have affected task performance in the course of the study. Before every session start, participants responded to the short version of the Positive Affect and Negative Affect Scale (PANAS; Watson et al., 1988) to assess current mood. Figure 6 depicts the average ratings for three different aspect of participants’ current mood with possible relevance to the learning process, namely, novelty/motivation (comprised the adjectives interested, excited, inspired, enthusiastic, determined; see Figure 6A), attention (comprised the adjectives alert, attentive, active; see Figure 6B) and frustration (comprised the adjectives distressed, downhearted, upset, frustrated, irritable; see Figure 6C). A linear regression analysis showed a downward trend in the rating of novelty/motivation (EG: *y* = −0.8X + 18.4, *R^2^* = 0.84; CG: *y* = −0.4X + 16.73, *R^2^* = 0.12) and attention (EG: *y* = −0.37X + 12.2, *R^2^* = 0.86; CG: *y* = −0.48X + 11.5, *R^2^* = 0.32) for both the EG and the CG with duration in the study. Also, we observed high correlations (Pearson’s correlation) between the percentage change in the BOLD signal for both target ROIs (i.e., bilateral anterior insula for EG and bilateral primary auditory cortex for CG) and the rating of novelty/motivation (EG: *r* = 0.73 and CG: *r* = 0.51) and attention (EG: *r* = 0.23 and CG: *r* = 0.67). In contrast, we observed a positive trend in the rating of frustration for both the EG (*y* = 0.41X + 4.2, *R^2^* = 0.67) and the CG (*y* = 0.05X + 5.1, *R^2^* = 0.017; see Figure 6D). The correlation between the percentage change in the BOLD signal for both target ROIs and the ratings of frustration, however, were negative for both the EG (*r* = −0.4) and the CG (*r* = −0.16). Thus, low motivation and reduced attention levels may have resulted in reduced self-regulation performance as the training progressed. Supporting this explanation is our finding that participants’ ability to up-regulate the anterior insula activity improved in the last session, in which the task was novel again since participants performed the self-regulation task along with the emotion perception task.

After every run, participants indicated what strategies they had used in order to control the thermometer bar. We explored these self-reports and found that the kind of strategies participants used did not differ across sessions in which they were more vs. less successful in up-regulation of brain activity. For example, participants in both groups reported the use of emotionally charged imagery during regulation blocks (e.g., thinking about a sick friend, an annoying colleague at work, spending quality time with family and friends, or engaging in hobbies). Participants did not appear to change their strategies when they experienced no up-regulation success, even though they had been instructed to change strategies if those did not result in positive feedback. During the neurofeedback training, older adults needed to assess their performance continuously based on the feedback provided to them and simultaneously change their strategy to improve up-regulation of brain activity. It is possible that lower levels of cognitive flexibility associated with age resulted in less ability of our older study participants (compared to young participants used in previous research) to switch from an unsuccessful to a more successful strategy.

Another reason for relatively less successful up-regulation in our older adults than is typically reported in the literature with young adults could be that the present study design used longer intervals between training sessions due to logistic reasons. All neurofeedback training sessions were conduct on the weekends and thus extended the total duration of the experiment to four weeks for six rtfMRI sessions. Thus, it is possible that the total amount of training that our participants received was sufficient, but that the interval between training sessions was too extended for full skill transfer.

Taken together, the results from our study support feasibility of the rtfMRI neurofeedback approach in healthy older adults. In addition to evidence of older adults’ ability to volitionally up-regulate targeted brain regions, we observed improvement in cognitive flexibility scores of older adults. The potential of this novel technique in aging research will be discussed next as will be challenges that this line of work has to overcome in future applications with older populations.

### Studying Healthy and Pathological Aging via rtfMRI Based Neurofeedback

There is ample evidence that alterations in structural and functional brain aging are associated with decline in cognitive function (Grady, 2012). The complexity of neural activity and cognitive functions, however, makes exact mapping between brain and behavior extraordinarily difficult, and so these relations remain largely speculative, although they are ultimately testable. Some of the proposed explanations for the decline in cognitive performance are age-related gray and white matter atrophy (Good et al., 2001), synaptic degeneration (Toth et al., 2012), low blood perfusion (Liu et al., 2012), and change in whole-brain connectivity (Ferreira et al., 2016) such as disconnectedness or dysfunctionality of brain networks (Tomasi and Volkow, 2012). As summarized above, several neurofeedback studies have shown that it is possible to obtain volitional control over a circumscribed region or networks of regions, with cognitive and behavioral effects (Shibata et al., 2011; Ghaziri et al., 2013; Ruiz et al., 2013a; Kim et al., 2015). In the rtfMRI approach, we constitute brain activity as the independent variable while cognition and behavior serve as the dependent variables. Thus, rtfMRI is a novel tool that can help us to test these proposed brain structural and functional explanations of cognitive decline in aging.

Aging is typically associated with overall decrease in gray matter. Some evidence suggests that the overall decrease in gray matter does not necessarily lead to cognitive decline; rather small decrease in gray matter in specific brain areas such as the insula (Good et al., 2001; Sowell et al., 2003), dorsolateral PFC (Grieve et al., 2005), and medial PFC (Uylings and de Brabander, 2002) may underlie age-related cognitive decline. A neurofeedback study on young adults showed significant increase in gray matter and white matter connectivity in areas related to attention (e.g., the intraparietal sulcus and the middle frontal gyrus) along with enhanced performance in visual and auditory attention after 13 weeks of neurofeedback training (Ghaziri et al., 2013). Thus, it is possible to increase gray matter in a particular brain area via neurofeedback training in young adults. The use of neurofeedback training in aging could similarly result in reduced rate of gray matter atrophy or even increase in gray matter in specific areas of the brain with possible effects on improved cognitive functioning in aging.

A number of studies have suggested that the aging brain has the potential to re-organize neural activity to compensate for anatomical and physiological change such as proposed in HAROLD (Cabeza, 2002) and PASA (Davis et al., 2008). This re-organization of brain activity is considered to be a compensatory mechanism by the aging brain to counter physiological deficit. In particular, these effects are observed in high-preforming when compared with low-preforming older adults in various domains of cognition such as episodic memory retrieval (Madden et al., 1999; Grady, 2002), episodic memory encoding (Logan and Buckner, 2001; Stebbins et al., 2002), working memory (Dixit et al., 2000; Reuter-Lorenz et al., 2000), perception (Grady et al., 1994; Grady, 2002), and inhibitory control (Nielson et al., 2002). Important questions that have not been answered yet are why some but not other older adults show this compensatory neural re-organization, and to what extent it is possible to train low-preforming older adults to use compensatory neural re-organization for performance improvement. As mentioned earlier, rtfMRI studies have demonstrated increase in connectivity between two brain areas or a network of regions using neurofeedback training (Koush et al., 2013; Kim et al., 2015). This raises the possibility that rtfMRI based neurofeedback training could be used in older adults to enhance compensatory neural re-organization towards performance improvement. This would importantly inform current models of aging and significantly advance scientific understanding of neural mechanisms in the aging brain.

There is evidence of some qualitative similarity between cognitive decline in pathological and in healthy aging, even though pathological and healthy brain aging differ in the rate and extent of the cognitive decrements (Walhovd et al., 2014). Both AD and Parkinson’s disease (PD) are characterized by memory difficulties, slowed processing speed, impaired attention, visuoperceptual/visuospatial dysfunction, and dysexecutive syndrome (Weiner et al., 2013; Todorova et al., 2014). Neuroimaging evidence further supports disturbed functional connectivity between the frontal and parietal lobes in AD patients (Wang et al., 2007; Zhang et al., 2010), and dysfunction of cortico-striatal functional connectivity in PD (Kwak et al., 2010). Neurofeedback training studies have reported improvement in memory (Berman and Frederick, 2009) and verbal comprehension (Becerra et al., 2012) in AD patients. These studies used EEG based neurofeedback. Recently rtfMRI based neurofeedback has been used to train PD patients. Patients learned to increase activity in the supplementary motor area (SMA) and subsequently improved their speed of finger tapping (Subramanian et al., 2011); but see Buyukturkoglu et al. (2013) for a contradictory finding. Thus, there is some initial evidence suggesting neurofeedback training success in pathological aging.

### Challenges in Using rtfMRI Based Neurofeeback in Aging Populations

Evidence of neurofeedback success in young adults and clinical populations summarized throughout this article, combined with the feasibility data of healthy older adults from our group, suggest that rtfMRI based neurofeedback may be a potential tool to study the aging brain and to inform development of interventions to maintain cognitive function and defray cognitive decline in older adults. This exciting new approach to the study of cognitive and brain aging, however, also faces challenges.

For example, in our feasibility study participants received contingent rtfMRI neurofeedback, but were only able to maintain moderate levels of BOLD up-regulation in the anterior insula or the primary auditory cortex, respectively. As discussed, possible explanations for these moderate levels of self-regulation found in our study may come from fatigue and lack of novelty when older adults engage in training sessions that are highly cognitively demanding. Future studies need to apply conditioning paradigms like shaping to improve self-regulation success (Peterson, 2004). In shaping, small changes towards the desired behavior are rewarded, which leads to gradual change across successive trials. Thus, the new method would change the baseline BOLD value after each TR so that any gradual change towards the desired BOLD signal value is rewarded. The dual-task conflict that may underlie relatively lower self-regulation performance in older adults also needs to be addressed in future studies in the attempt to reduce the cognitive demand in neurofeedback training. One alternative could be to reduce the frequency at which feedback is presented, as this would reduce the overall cognitive load of continuous evaluation of feedback. Also, due to logistic reasons in our feasibility study, we had to conduct the neurofeedback training on the weekends. This extended the total duration of the experiment to a couple of weeks for six rtfMRI sessions, which is longer than a typical rtfMRI study. Thus, it is possible that the total amount of training that our participants received was sufficient, but that the interval between training runs was sub-optimal for learning.

The issue of slow learners, which may particularly apply to older adults, can be addressed in future research by increasing the number of training sessions, e.g., 13 weeks of neurofeedback training (Ghaziri et al., 2013). However, greater numbers of training sessions will drastically increase the cost of conducting a study to the extent where this approach would not be feasible anymore as clinical intervention given the high costs for MRI. Therefore, development of less cost-intensive neurofeedback training methods based on cheaper modalities such as EEG or fNIRS is crucial. However, each of these alternative modalities comes with a set of limitations. For example, both EEG and fNIRS cannot be used for self-regulation of deeper brain region (e.g., anterior insula, amygdala), which are particularly relevant for emotion processing and thus will limit domains of study for these techniques. A useful approach for future research is to start neurofeedback training with rtfMRI and later transition to cheaper modalities. Along these lines, a technique called “EEG Finger-Print” was developed (Meir-Hasson et al., 2014). In this approach, advanced signal processing to remove artifacts and machine learning algorithms are applied on EEG data acquired simultaneously with fMRI to find EEG features that can predict specific deeper brain activity. With this approach, an experiment can be designed in which older adults initially learn self-regulation of a circumscribed brain region or network of brain regions by using rtfMRI based neurofeedback training. During the rtfMRI training, simultaneous EEG recordings will be conducted to determine the neuroelectric components that correlate with the volitional control of the ROIs. Using the EEG Finger-Print technique, the EEG neural-correlates of volitional control of deeper brain region could be identified. The identified EEG pattern can be used to continue neurofeedback training via EEG without fMRI. This would make long neurofeedback training studies cost-effective and more flexible (i.e., portable, convenient system). The EEG Finger-Print technique could also target older adults who have implanted stents, pacemakers, or other metallic implants in their body and hence are unable to participate in MRI experiments. This would allow test of a more representative sample of older adults.

Another factor that could influence learning in older adults pertains to the way instructions are given and the type of feedback. According to Knowles’ theory of adult learning (Knowles, 1984), older adults learn better if they are aware of the background of the topic. Also, readiness to learn and motivation to attain new information are critical factors, which is in line with the current study showing that lower motivation levels reduced the performance in self-regulation. Therefore, in future studies, it will be important to instruct older adults in a way that keeps them motivated and provides them with background information pertaining to the study. For example, information can be presented to older adults in a manner that is personally relevant to them (Zurakowski et al., 2006). In this context, it is also necessary to explore new ways of providing feedback apart from traditional feedback modalities such as the use of virtual reality where feedback can be associated with certain events in the virtual world and may thus be more intuitively processed and eventually more effective.

## Conclusion

Age-related cognitive decline is of increasing societal, political, and economic concern, and dramatically affects individual lives. Improvement of neuroimaging techniques has advanced the investigation of cognitive decline in aging with a particular focus on brain processes underlying age-related change. Although this research field has benefited greatly from recent advancements in imaging technology, there are still a number of unresolved issues such as pertaining to age-related change in brain structure and function underlying inter-individual variation of cognitive decline in aging or validation of proposed theories of aging related to loss of gray matter in certain regions of the brain as well as hypoactivation of brain areas or networks. We propose that rtfMRI neurofeedback offers a potent tool to study cognitive decline processes towards development of effective training and intervention protocols in aging. Preliminary results of our feasibility study suggest that it is possible for older individuals to volitionally control a circumscribed brain area and that neurofeedback training of anterior insula is associated with increased cognitive flexibility, supporting benefits of this technique in use with older adults. However, this nascent field faces some challenges that need to be overcome for advanced application in aging. We hope that this article will spur research in unexplored areas of cognitive aging, towards development of effective intervention programs to promote cognitive health in older adults.

## Author Contributions

MR: design of the work, data acquisition, fMRI analysis and writing the article. AQV: design of the work, data acquisition, behavioral analysis. AD: data acquisition, fMRI analysis. RAC: final approval of the manuscript. RS and NCE: design of the work, interpretation of results and final approval of the manuscript.

## Funding

This work was supported by the Center for Cognitive Aging and Memory, Clinical Translational Research Program (CAM-CTRP) in the Institute on Aging, the Department of Psychology and the Department of Biomedical Engineering at University of Florida.

## Conflict of Interest Statement

The authors declare that the research was conducted in the absence of any commercial or financial relationships that could be construed as a potential conflict of interest.

## Footnotes

^1^http://www.aarp.org/health/brain-health/

^2^http://www.alz.org/publichealth/2013-report/index.html

^3^http://brainfoundation.org.au/healthy-brain
